# Temptation as a key driver between affective states and usage outcomes of problematic usage of the Internet: A 14-day ambulatory assessment study

**DOI:** 10.1371/journal.pone.0352776

**Published:** 2026-07-29

**Authors:** Andreas Oelker, Anna Knorr, Matthias Brand, Silke M. Müller, on behalf of the FOR2974 Consortium

**Affiliations:** 1 General Psychology: Cognition, Faculty of Computer Science, University of Duisburg-Essen, Duisburg, Germany; 2 Center for Behavioral Addiction Research (CeBAR), Center for Translational Neuro- and Behavioral Sciences (C-TNBS), University Hospital Essen, University of Duisburg-Essen, Essen, Germany; 3 Erwin L. Hahn Institute for Magnetic Resonance Imaging, Essen, Germany; Methodist University Cape Fear Valley Health School of Medicine, UNITED STATES OF AMERICA

## Abstract

Theoretical models of problematic usage of the Internet (PUI) assume that stress and negative mood can trigger cravings and desires as central drivers to use certain online applications that are expected to provide pleasure and relief, which increases the likelihood of use. Learning and reinforcement mechanisms can result in increased and uncontrolled Internet use, that manifests itself in the form of, for example, neglecting important areas of life like relationships or work. To test these associations in a natural environment, we conducted a 14-day ambulatory end-of-day assessment together with a diagnostic interview at baseline to identify different severity levels of PUI. The sample (N = 900) was part of a multi-center study (FOR2974) including participants who engaged in at least one of the following online activities: gaming, pornography use, social network use, or buying-shopping in either a non-problematic (*n* = 419), risky (*n* = 242), or pathological (*n* = 239) way. The adherence rate was about 88%. The data were analyzed using ANOVAs, generalized linear mixed modeling (GLMM), and multigroup multilevel structural equation modeling (MG-ML-SEM). In participants experiencing more severe PUI, ANOVAs revealed greater neglect of other areas of life (*f* = 0.531), longer use times (*f* = 0.442), and more stress (*f* = 0.220–0.248) as well as worse mood in daily life (*f* = 0.220–0.237). Overall, the variables and random effects in the GLMM explained *R²*_conditional_ = 79.9% (*R²*_marginal_ = 22.6%) of variance in the prediction of daily usage with temptation as primary within-person effect (*OR*=2.53) and PUI severity as between-person effect (*OR* = 0.41 to 11.33) being the main predictors. Results of the MG-ML-SEM indicate that pleasure and relief are reinforcing factors for continued use of the Internet with temptation as the central main driver (strongest effect sizes), as theoretically expected. With the extensive sample, the longitudinal approach, and the robust statistical methods, it shows that assumptions of common theoretical models for PUI can also be found in everyday life.

## Introduction

Problematic usage of the Internet (PUI) is a growing field of concern and is often defined as an excessive use of online applications, which leads to functional impairment and/or marked distress [1]. PUI is also addressed in the ICD-11 as several types of behavioral addictions with structured diagnosis have been included which may occur predominantly online. Included ICD-11 categories are gaming disorder, gambling disorder, and other unspecified or specified disorders due to addictive behaviors [2]. Other disorders due to addictive behaviors may include problematic pornography use, social network use, and buying-shopping, as these types of PUI have clinical relevance, theoretical embedding, and empirical evidence [3,4]. According to the ICD-11, gaming disorder, like other types of PUI that fall into the category of behavioral addictions, is characterized by marked distress and functional impairments in daily life due to (a) difficulties in controlling the behavior, (b) increasing neglect of other activities and associated prioritization of the behavior, and (c) continuation of the behavior despite negative consequences [2].

Theoretical (etiological) models of PUI [5–8] hypothesize associations between experienced affective states (e.g., stress and mood) and emerging temptations or cravings to use the Internet, which result in recurrent use of specific online applications and respective usage outcomes, such as experienced pleasure, relief, and neglect of alternative activities due to Internet usage. According to the I-PACE model and recent interpretations [7,9], these effects may vary depending on the severity of PUI symptoms. For example, feelings of pleasure and gratification are assumed to be already important in the early stages of the development of PUI, whereas the experience of compensation or relief gains more relevance in later stages, even though the relative importance of gratification and compensation may fluctuate intra-individually [9]. Research on PUI has increased dramatically in the past decade; however, most empirical findings are based on cross-sectional data. Such findings from cross-sectional studies on PUI consistently demonstrate that higher symptom severity is associated with heightened temptations or urges to use [10], heightened experience of pleasure and/or relief through the use [11,12], but also with negative mood or impaired psychological well-being [13–15] and feelings of (chronic) stress [16,17]. What is still rare – but urgently needed – is research into these connections across the different types of PUI in a non-cross-sectional approach [18], because cross-sectional designs are limited to between-person differences at a single time point, whereas for example, ambulatory assessments capture dynamic intraindividual processes in everyday life and help to disentangle within- and between-person effects.

### Mechanisms of PUI in everyday life

Studies have shown that unpleasant situations may trigger internal conditions like stress or negative mood and that for some people the use of the Internet is a strategy to cope with these negative affective states, e.g., gaming after a stressful workday or scrolling on social media after a conflict with a friend [19–21]. Through the use of the Internet, pleasure and relief from negative feelings may be experienced; also called the experience of gratification and compensation [11,12]. The repeated experience of gratification and compensation through usage of the Internet is assumed to drive explicit and implicit learning mechanisms, which manifest in specific motives, beliefs or expectations, e.g., that the behavior helps to cope and regulate emotions [22]. Thus, when a new perceived negative situation is faced, urges and cravings to use the Internet are triggered, which have been shown to increase the probability of (problematic) Internet use [23–25]. Regardless of whether the previous learning is explicit or implicit, it is assumed that the triggers will be linked closer to the Internet use, stronger stimulus-response associations and habits are created, as has been shown recently using a pavlovian-to-instrumental transfer paradigm in the context of gaming [26]. In accordance with theories on drug addiction [27,28], this habit formation can lead to an automated behavior that may interfere with other responsibilities that are neglected, like work, school, or social relationships. These mechanisms have also been addressed and summarized in the I-PACE model and its current interpretation [6,7,9].

To test if the theoretical assumptions and cross-sectional findings can also be applied to everyday Internet use in a natural environment, other assessment methods need to be carried out. Using ambulatory assessments, cognitive and affective states and behavior enactment can be assessed in a natural environment. As multiple data points per person are measured, the underlying dynamics of PUI can be analyzed in depth and predictions of future behavior enactment can be derived [18,29]. In the following, we will address each of the aforementioned connections in the context of PUI and daily life. In particular the connections of mood and stress, leading to heightened temptation to use, which increases usage and resultant outcomes such as perceived pleasure, relief, and neglect of other activities.

### Results of ambulatory assessment studies in the context of PUI

Ambulatory assessments have been used in PUI research for several years with increasing frequency. However, the studies vary greatly, e.g., regarding the investigated type(s) of PUI, procedures, time frames, and measures. In the following, we will aim to bring these heterogeneous methods together in order to draw conclusions for each of the above-mentioned associations.

The research on stress in PUI is especially heterogeneous concerning definitions of stress and the implemented methodologies. Therefore, it is important to distinguish between chronic and acute stress. In this study, we focus on acute stress, which is the short-term experience of stress, but could be influenced by chronic stress in the form of a daily higher stress level due to a permanent stressor [30]. As a state variable, acute stress is particularly suitable for an ambulatory assessment. Overall, most studies show a positive correlation between stress and PUI symptom severity as for example in a scoping review for buying-shopping [31], in general Internet use [21], in a longitudinal study (assessment every six months for three years) for gaming and gambling [32], in an experimental study on social network use with a stress induction [33], and in an experimental study on pornography use [34]. Literature addressing the direct effects of perceived stress on PUI in a daily context is scarce. A two-week study about gaming abstinence showed that there is lower daily stress compared to a control group, whereas stress was considered as outcome variable of gaming disorder and not as a trigger [35]. A seven-day study about the influence of parental stress found no effects on the children’s screen time [36]. Furthermore, in a 14-day study, it was shown that the effect of stress on problematic phone use was fully mediated by negative emotions [37].

Sometimes stress and (negative) mood are used synonymously, with mood appearing to be a broader term that can include stress in the sense of a general negative emotional state. Mood and mood modification are considered important for PUI, for example as a component of addiction [38]. Mood modification is also included in the cognitive-behavioral model of pathological Internet use [39] and in the I-PACE model [6,7,9]. These models describe regulating negative mood or coping with adverse emotions is a central driver for repeated use of online applications. Empirical findings confirm that online applications are used for mood regulation, e.g., for pornography use [40], general Internet use [41], or in a meta-analysis on gaming disorder [42]. In one ambulatory assessment study [43] it was found, that for buying-shopping, daily stress and mood do not predict buying-shopping episodes. However, negative mood prior to shopping episodes decreased after the episode [43]. In another study the link between negative mood and pathological buying-shopping could be found [44]. In a 14-day study about gaming, it was found that negative emotions of parents lead to an increased gaming behavior of their children on between- and within levels. More positive mood states of the parents did not influence the gaming behavior. Although the children’s emotional states were also measured, the effects were not reported [45]. In another study, the parental mood did not predict the children’s screen time [36]. Mood as an outcome of PUI has been found for problematic pornography use, where binge episodes led to a decreased mood, increased stress, and anxiety [46]. Thus, the daily findings of mood and stress seem to be mixed.

Mood, feelings of craving and temptation to use online applications are closely related. The anticipated mood regulation (in terms of expectations about experienced pleasure and/or relief through the use) is assumed to promote the emergence of urges and desires to use the Internet [7,9]. Similar to other types of addictive behaviors [47], empirical findings indicate that craving is a central element of PUI, e.g., for problematic pornography use [48], for problematic online buying-shopping [49], for gaming disorder [50,51], for gambling disorder [52], and problematic social network use [53]. The latter study also showed that craving acts as a mediator between psychological distress and problem severity. Similar results were found in an ambulatory assessment in the context of problematic gambling, where affect did not predict the gambling behavior, but the desire to gamble, which in turn predicted the gambling behavior. These effects were only found for negative affect and not for a positive mood [54]. Regarding gaming disorder, higher symptom severity was associated with more craving in daily life and more time spent gaming [55]. Similar results were found for gambling [56] and for social media use [57].

Looking at direct outcomes of Internet use, pleasure and relief or gratification and compensation are frequently mentioned [6,7,12,58]. As mentioned earlier, the experience of gratification (pleasure) is said to be highly important in the early stages of behavioral addictions and to remain important throughout the course of the addiction, whereas the experience of compensation (relief) may occur additionally, especially in later stages [9]. Consistently, it has been shown empirically, that individuals with pathological Internet use (across several types of PUI) report more compensation through the Internet usage than individuals with a risky use, and these in turn report more compensation than individuals with a recreational use. Gratification however, seems to be high for all classifications of symptom severity, but the experience of pleasure could vary depending on the specific type of PUI [11,59]. In a 28-day ecological momentary assessment, it was shown that positive outcome expectancies alone did not predict gambling behavior, but that pleasure together with craving and other factors was a determinant for gambling behavior [60]. In a 6-week ecological momentary assessment, relieving effects through episodes of problematic Internet use were found [61]. In sum, pleasure and relief seem to be important outcomes of daily Internet use and might differ depending on the symptom severity of PUI.

Another (negative) outcome of Internet use is the neglect of alternative activities. Frequent neglect of other important areas of life such as relationships or interferences with work or school are important for the diagnosis of gaming disorder [2]. Besides an overall worse quality of life through PUI [62], neglects of important facets in life may cause marked distress. There are reports about sleep deprivation because of gaming leading to depressive symptoms [63], neglect of personal hygiene, hobbies, socializing, work, or school responsibilities due to gaming [64], neglect of responsibilities and a lack of control of emotions due to pornography use [65], neglect of work and decreased job performance due to problematic social network use [66] and worsened academic performance due to problematic social network use [67]. It has also been shown that a social media detox leads to improved mood, sleep, and productivity [68]. In a 14-day ecological momentary assessment study, financial and relationship harms through gambling (sports betting) were found [69]. Daily problematic smartphone use showed negative effects on satisfaction with life through the interruption by the person’s smartphones [70]. As found in a systematic review for gaming, the negative impacts and neglect of alternative activities in the context of PUI in daily life need to be researched more closely as there are unclear findings in current longitudinal studies [71].

All these unclear results in the daily context prompted us to conduct the present study, in which we sought to answer the research questions: 1a. *Are daily stress, daily mood and daily temptation predictors of the daily engagement in an online activity?* 1b. *Do these effects vary for different levels of symptom severity of PUI?* and 2a. *To what extent do daily pleasure, daily relief and daily neglect depend on daily mood, daily stress, daily temptation, and daily use time?* 2b. *Do these effects vary for different levels of symptom severity of PUI?*

**Fig 1:**
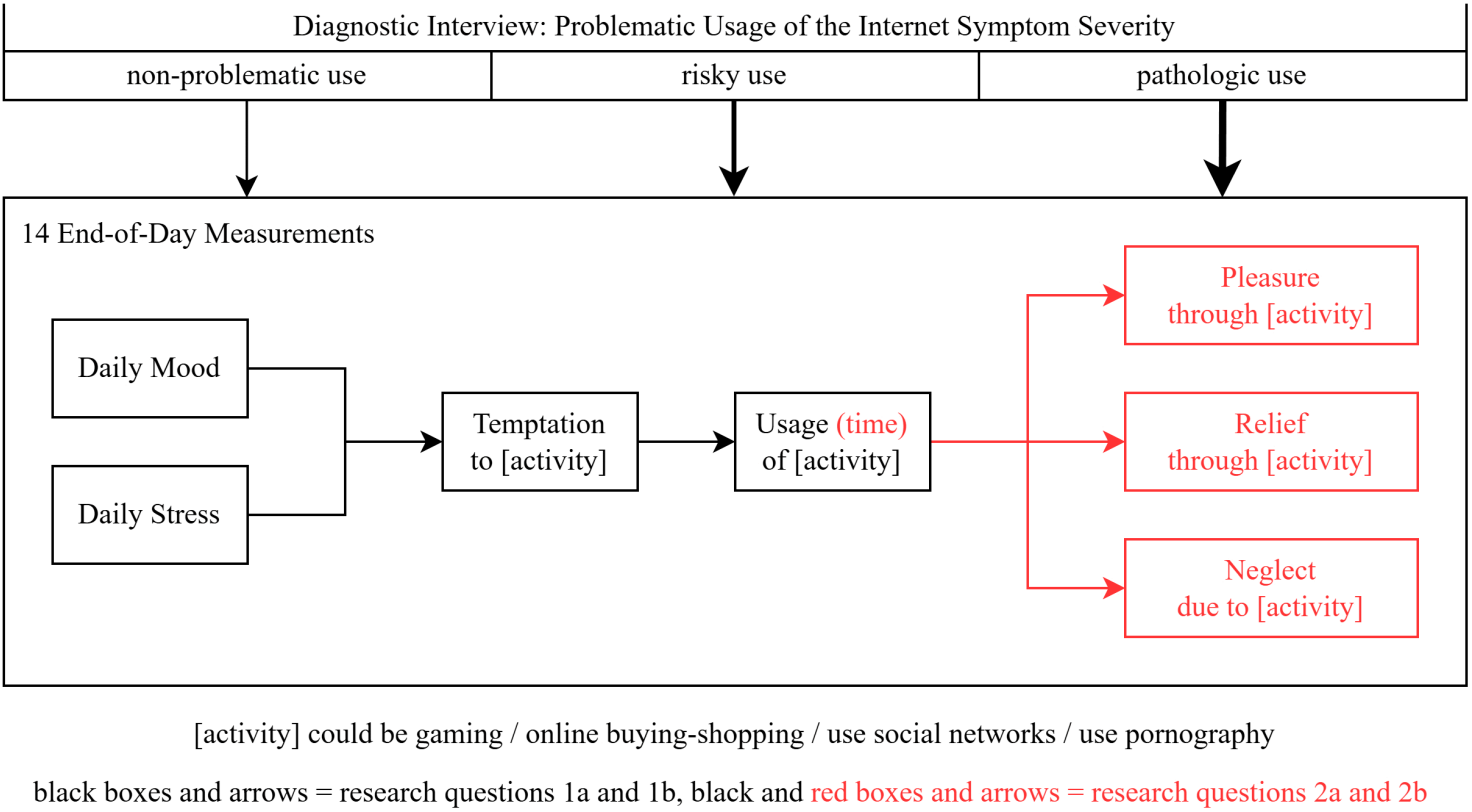
Theoretical model of daily processes within problematic usage of the Internet. With higher symptom severity of PUI the connection between the emotional triggers of stress and mood are expected to grow stronger to the subsequent temptation to use the Internet. The anticipation of pleasure and relief amplifies this process and with an increased use time more pleasure and relief are expected, but other important duties (e.g., work or relationships) are neglected.

To answer these questions, we tested our theoretical model (Fig 1) with empirical data from a 14-day ambulatory end-of-day assessment in combination with a previous classification of the severity of PUI symptoms based on a diagnostic interview. We hypothesize that people tend to have greater temptation to use the Internet and actually use the Internet more on days with a negative mood and a high stress level. Furthermore, we expect that people experience pleasure and relief through the use of the Internet, especially when the temptation is high, but that they also neglect other activities because of the increased use time. With a higher symptom severity, the described effects are expected to be stronger (i.e., non-problematic use < risky use < pathological use).

## Methods

The standardized diagnostic interview for PUI symptom severity and the ambulatory assessment applied in the present study were part of the sub-project RP1 (for details see https://osf.io/6x93n/) of the multi-center DFG-funded research unit FOR2974 [72]. The complete test battery can be viewed in the OSF. The recruitment included people with non-problematic, risky, and pathological use of the PUI types gaming, buying-shopping, social network use, and pornography use. The full “core battery” consisted of a telephone interview for recruitment and screening purposes, an extensive laboratory assessment including a standardized diagnostic interview, questionnaires, paradigms, and neurocognitive tasks, a subsequent 14-day ambulatory end-of-day assessment, and a six-month follow-up survey. The data collection was conducted in a standardized manner at various recruitment sites (universities and clinics) in Germany (for more details on the research unit see https://osf.io/n5cd7/). For the present study, only the standardized diagnostic interview and the ambulatory assessment were used for the analysis. In line with the I-PACE model, this study takes a transdiagnostic approach to PUI, analyzing the different types of PUI (gaming, buying-shopping, social network use, and pornography use) in an aggregated manner focusing on the common mechanisms. Results of the ambulatory assessment have not been published previously in an aggregated manner, i.e., across all investigated types of PUI. Results on other parts of the laboratory assessment (aggregated as well as in different sub-samples) have been published elsewhere [11,73–76].

### Sample

The sample is part of the first cohort of the FOR2974 multi-center study, including participants who completed the laboratory study and completed at least one day of the ambulatory assessment from October 2021 through August 2024. Exclusion criteria for recruitment and analysis were learning or developmental disorders, existing psychosis, substance use disorders, and consumption of any psychoactive substances. Inclusion criterion was at least an occasional use in the last 12 months of one of the following: gaming, pornography use, social network use, or online buying-shopping. The current final sample comprises *N* = 900 participants with ages between 18 and 65 years (*M* = 26.67, *SD* = 7.872). Of those, 482 (53.56%) participants were male, 413 (45.89%) female, and 5 (0.55%) indicated a non-binary or other gender. Concerning PUI type and classification, 419 (46.56%) participants were classified with a non-problematic use, 242 (26.89%) with a risky use, and 239 (26.56%) with a pathological use and were assigned to the types of gaming *n* = 386 (42.89%), buying-shopping *n* = 255 (28.33%), social networks *n* = 138 (15.33%), and pornography *n* = 121 (13.44%). To be assigned to a PUI type, people had to have at least an occasional use of the application in the past 12 months, but could also use other applications. Further descriptive statistics of the study sample are shown in the supporting information (see S1–S6 Tables).

### Diagnostic interview

To classify participants into the groups of non-problematic, risky and pathological Internet use, a standardized diagnostic interview (AICA-SCI:IUD adapted from the AICA-SKI:IBS; [77]) was applied at the beginning of the laboratory assessment. The AICA-SCI:IUD is based on the nine DSM-5 criteria for gaming disorder [78] supplemented by additional questions about functional impairments in daily life and was adapted for each PUI type. If no more than one criterion was fulfilled, the behavior was classified as non-problematic, with two to four fulfilled criteria the behavior was classified as risky, and was classified as pathological with a minimum of five fulfilled criteria. The interviews were conducted by trained doctoral and master’s students and supervised by experienced clinicians. In addition, a regular exchange took place to ensure uniform classification which was supervised by licensed psychotherapists.

### Ambulatory assessment

The ambulatory assessment started the day after the laboratory testing and lasted for 14 consecutive days. It was conducted as an end-of-day assessment, with the participants receiving automatic invitation links via email at 6 p.m. each day. Participants had time until 10 a.m. the next morning to complete the daily online survey in their preferred browser and device. After that, the corresponding invitation links expired and later participation was no longer possible for that day. The daily surveys were split into two parts.

The first part consisted of questions about the day in general. Participants answered each of the following questions on a 10-point Likert scale: *How was your mood today?* (from 0 = *very bad* to 9 = *very good*), *How stressed did you feel today?* (from 0 = *not stressed at all* to 9 = *very stressed*), *How strong was the temptation to [game/use pornography/…] today?* (from 0 = *not strong at all* to 9 = *very strong*) and, in addition, *Did you [game/use pornography/…] today?* (with a binary *yes/no* answer format). We define mood here as a subjective affective state characteristic of the day in question, which can range from very negative to very positive, and which is not necessarily tied to a specific event or object. Stress, however is a state of arousal that arises when individuals perceive environmental demands as taxing or exceeding their (adaptive) resources, e.g., motivation or time. Accordingly, the stress rating in our ambulatory assessment represents the subjectively perceived stress level for the respective day. Temptation is to be interpreted as a motivational state characterized by the perceived attraction of the online activity and the difficulty of resisting it with a hedonistic focus. Accordingly, the temptation item represents the subjective strength of the temptation or urge to engage in the behavior felt on that day – regardless of whether the behavior was ultimately carried out or not.

The second part then proceeded with specific questions about the usage of the Internet application. If the previous question about the usage was answered with “*yes”*, the participants had to first indicate their use time in hours and minutes on that day. If they answered previously with *no*, the participants had to provide an alternative recreational activity (not work/school related) that was done on the day, to keep the number of items per survey equal and decrease the risk of bias as the ambulatory assessment was remunerated at 5€ per day of participation. The following questions were the same for both answers (*yes* and *no*), differing only in that either the recreational activity or the respective online activity was included in the questions. The questions were (with answer format 10-point Likert scales): *How strong was the feeling of pleasure when you [were gaming/used pornography/…]?* (from 0 = *not strong* at all to 9 = *very strong*), *How strong was the feeling of relief when you [were gaming/used pornography/…]?* (from 0 = *not strong at all* to 9 = *very strong*) and *How strong have you neglected other activities due to [gaming/pornography…]?* (from 0 = *not strong at all* to 9 = *very strong*). We define pleasure as the perceived strength of positive affective or gratifying experiences arising from the online activity for a specific day. Relief can also be defined as a positive feeling, but one that arises from the removal or (partial) compensation of negative affective states such as distress or tension through the online activity. Neglect of other activities are defined as the subjectively perceived extent to which alternative (not further specified) actions or duties were reduced or omitted due to the online activity. This might include neglecting work, study or homework, but also neglecting more basic activities like eating, drinking, sleep, social contact, or hygiene.

### Analysis procedure

The analysis consists of the descriptive statistics and comparisons between the classified groups (non-problematic, risky and pathological use) using ANOVAs calculated with SPSS version 29. Bonferroni correction was applied for post-hoc pairwise comparisons. In case of variance inhomogeneity, the Welch-Test was calculated with post-hoc Games-Howell comparisons. Furthermore, we used multilevel models for the analysis of the longitudinal data. The data from all participants were aggregated, i.e., analyses were performed across different PUI types. The lme4 and lavaan packages for R were used for the calculation of the multilevel models.

To answer the research questions 1a and 1b, we analyzed the first part of the ambulatory assessment with all complete end-of-day surveys (within-person) together with the classification (i.e., non-problematic, risky, or pathological use) from the diagnostic interview (between-person), see Fig 1 (black part). The analysis for the research questions 2a and 2b comprised the classification (between subjects) as well as the first and second part of the ambulatory assessment (within subjects) – but only for days on which the Internet application was used – see Fig 1 (black and red part). We decided to only use the active days for research questions 2a and 2b, because these questions concerned the effects the Internet use had on subsequent outcome variables, which can only be derived from days on which the behavior was actually carried out.

For simple between-group comparisons (ANOVAs), we calculated mean values per person across all days (of participation or usage) for each of the variables of the ambulatory assessment, i.e., *mood, perceived stress, temptation to use, use time in hours, experienced pleasure, experienced relief, neglect of other activities*. We used Cohen’s *f* for effect sizes, as this value is also suitable for variance tests where the independent variable has no homogeneity of variance (Welch-Test).

Concerning the multilevel analysis, we calculated a generalized linear mixed model (GLMM) for research questions 1a and 1b (with a binary outcome) and multigroup multilevel structural equation modeling (MG-ML-SEM) for research questions 2a and 2b (with multiple continuous outcomes on days with indicated usage). To prepare the data for the multilevel analysis, all predictors (*mood, stress, temptation, use time*) were *centered within clusters* (cwc), also called *group mean centering*, for the corresponding (sub-)dataset, i.e., the individual person’s mean was subtracted from each person’s time point value. This enables a better interpretation of the within-person (level 1) effects and reduces the interference from between-person effects [79]. Congruently, as the MG-ML-SEM requires an additional between (level 2) model, the predictors and mediators were *centered at the grand mean* (cgm), i.e., the overall mean of each variable across all participants was applied to the clusters, to interpret pure between effects.

The GLMM has a binary output (usage = yes/no) with a logit link, i.e., the output represents the probability of usage. It uses the maximum likelihood estimator with a Laplace approximation and bound optimization by quadratic approximation (BOBYQA). We calculated a random intercept, a random slope, and a cross-level interaction model and chose the best fitting model based on fit indices. We chose random intercept models because they offer advantages in terms of convergence. In comparison, random slope models allow statements to be made about the strength of the predictors, which can also be achieved in part by cross-level interactions in random intercept models. In this study, the moderator symptom severity is of particular interest, which is why we calculated cross-level interactions with random intercepts.

For the MG-ML-SEM, we calculated the intraclass correlations (*ICC*) for the null model of each dependent variable to see if a multilevel structure was needed and continued with the implementation of the theoretical and initial model on level 1 (classification of symptom severity) and level 2 (mood, stress, temptation, use time, pleasure, relief and neglect). After implementing the initial model, we made adjustments to the model based on the resulting data – that is, using model fit and modifier indices >10 – in combination with theoretical considerations. Because of the heterogeneity of variance of the dependent variables, we calculated multigroup (non-problematic, risky, and pathological) SEMs for a better understanding of the between effects of symptom severity [80]. As an estimator we used the maximum likelihood (ML). The optimization method was the nonlinear minimization subject to box constraints (NLMINB).

### Ethics

All applied procedures were in accordance with the ethical standards of the responsible committee on human experimentation (institutional and national) and with the Helsinki Declaration of 1975, as revised in 2000 (5). Informed consent was obtained from all participants to participate in the study. The overall study protocol was approved by the local ethics committee of the University of Duisburg-Essen (ID: 1911APBM0457). Participants’ data were pseudonymized and stored in accordance with the General Data Protection Regulation of the European Union using the ALIIAS framework [81].

## Results

### Descriptive statistics

With a sample of *N* = 900 and an average participation of 12.38 days (out of 14 days) resulting in 11,139 complete end-of-day surveys and an adherence rate of 88.41%, we collected a robust dataset to address our research questions. For days on which the applications were used (subset), there are 5,410 complete end-of-day surveys with an average of 6.01 days of use per participant. Overall, 118 participants (87 with a non-problematic, 15 with a risky and 16 with a pathological use classification) had zero days of usage. The values of the number of days of participation are left skewed and leptokurtic (compare *Sk* and γ₂ of Table 1), which indicates again a high adherence rate.

**Table 1:** Descriptive statistics and group comparisons of the variables assessed in the first part of the 14-day ambulatory assessment averaged over all days of participation.

	PUI group																							
	Non-problematic use (np, *n* = 419)						Risky use (ri, *n* = 242)						Pathological use (pa, *n* = 239)						ANOVA (N = 900)					
Variables	Min	Max	*M*	*SD*	*Sk*	*γ₂*	Min	Max	*M*	*SD*	*Sk*	*γ₂*	Min	Max	*M*	*SD*	*Sk*	*γ₂*	*F*	*df1*	*df2*	*p*	*f*	Post-hoc
days participated	1.00	14.00	12.64	2.347	−2.52	7.05	1.00	14.00	12.22	2.771	−2.13	4.33	1.00	14.00	12.07	2.917	0.00	3.74	4.228*	2.00	480.56	.015	0.133	np > pa
mood	2.23	9.00	5.81	1.138	−0.72	0.37	1.54	8.67	5.50	1.132	−0.24	0.67	0.80	9.00	5.14	1.216	−0.09	1.31	25.31	2.00	897.00	<.001	0.237	np > ri > pa
stress	0.00	7.00	3.18	1.314	0.07	−0.22	0.00	6.36	3.37	1.279	−0.08	−0.45	0.00	9.00	3.89	1.436	0.04	0.37	21.55	2.00	897.00	<.001	0.220	pa > ri,np
temptation	0.00	7.36	1.95	1.673	0.78	0.01	0.00	9.00	3.44	1.630	0.22	−0.26	0.00	9.00	4.17	1.797	−0.08	−0.16	144.49	2.00	897.00	<.001	0.568	pa > ri > np
days with usage	0.00	14.00	4.95	4.662	0.69	−0.83	0.00	14.00	6.14	3.843	0.29	−0.85	0.00	14.00	7.75	4.677	−0.10	−1.32	27.621*	2.00	530.34	<.001	0.323	pa > ri > np

Note. *No homogeneity of variance, the Welch-Test was calculated with post-hoc Games-Howell comparisons. With homogeneity of variance Bonferroni post-hoc comparisons were calculated.

### Group comparisons on averaged outcome variables (ANOVAs)

In general, individuals with a pathological use filled out less surveys (*M* = 12.07) than people with a non-problematic use (*M* = 12.64). Individuals with a risky use (*M* = 12.22) however, did not differ significantly from the other groups (see Table 1). Further, the pathological use group reported, on average, worse mood (*M* = 5.14), more stress (*M* = 3.89), and a higher temptation (*M* = 4.17) to use the online activity than the other two groups. The risky use group indicated worse mood (*M* = 5.50) and higher temptation (*M* = 3.44) than the non-problematic group (*M*_mood_ = 5.81; *M*_temptation_ = 1.95), however, differences in perceived stress were not significant between the two groups (*M*_ri_ = 3.37; *M*_np_ = 3.18; see Table 1). Concerning the days with usage of the preferred application, the number of days differs significantly between the three groups, with incremental increases from lower, medium, to high symptom severity for PUI (*M*_np_ = 4.95; *M*_ri_ = 6.14; *M*_pa_ = 7.75; see Table 1).

The group differences for stress, mood, and temptation are similar when only the days of usage are analyzed (see Table 2). The non-problematic use group had the lowest use times (*M* = 1.49), the risky use group was between the other groups (*M* = 2.15), and the pathological use group had the highest use times (*M* = 2.68) with a leptokurtic kurtosis (*γ₂* = 2.52), meaning when an application was used, it was used in large amounts.

**Table 2:** Descriptive statistics and group comparisons of the variables assessed in the first and second part of the 14-day ambulatory assessment averaged over all days with usage of the respective online activity.

	PUI group																							
	Non-problematic use (np, *n* = 332)						Risky use (ri, *n* = 227)						Pathological use (pa, *n* = 223)						ANOVA (N = 782)					
Variables	Min	Max	*M*	*SD*	*Sk*	*γ₂*	Min	Max	*M*	*SD*	*Sk*	*γ₂*	Min	Max	*M*	*SD*	*Sk*	*γ₂*	*F*	*df1*	*df2*	*p*	*f*	Post-hoc
mood	1.00	9.00	5.66	1.390	−0.18	0.27	1.80	8.75	5.29	1.242	−0.19	0.04	0.80	9.00	4.95	1.418	−0.15	0.84	18.84	2	783	<.001	0.220	np > ri > pa
stress	0.00	8.00	3.08	1.626	0.24	−0.25	0.00	6.60	3.32	1.445	0.05	−0.56	0.00	9.00	4.02	1.706	0.19	0.28	24.11	2	783	<.001	0.248	pa > ri,np
temptation	0.00	9.00	3.76	2.029	0.33	−0.22	0.00	9.00	5.02	1.737	−0.19	−0.07	0.33	9.00	5.25	1.892	−0.07	−0.45	47.950*	2	493.893	<.001	0.441	pa,ri > np
use time	0.03	5.95	1.49	1.272	1.14	0.74	0.08	8.56	2.15	1.673	1.11	1.13	0.17	10.73	2.68	1.719	1.19	2.52	42.998*	2	440.726	<.001	0.442	pa > ri > np
pleasure	0.00	9.00	4.68	1.849	−0.07	−0.31	0.67	9.00	5.28	1.648	−0.21	−0.18	0.00	9.00	4.87	1.875	−0.08	−0.23	7.43	2	783	<.001	0.139	ri > pa,np
relief	0.00	9.00	3.22	2.086	0.33	−0.50	0.00	9.00	4.21	1.904	0.07	−0.19	0.00	9.00	4.49	1.911	−0.07	−0.06	31.419*	2	492.312	<.001	0.357	pa,ri > np
neglect	0.00	8.00	1.43	1.507	1.34	1.96	0.00	7.56	2.33	1.714	0.75	0.35	0.00	9.00	3.14	1.984	0.35	−0.35	63.980*	2	453.644	<.001	0.531	pa > ri > np

Note. *No homogeneity of variance, the Welch-Test was calculated with post-hoc Games-Howell comparisons. With homogeneity of variance Bonferroni post-hoc comparisons were calculated. Use time is measured in hours.

Further, the risky use group indicated the most pleasure (*M* = 5.28) through the preferred application, whereas the pathological use group (*M* = 4.87) reported no significant differences in pleasure to the non-problematic use group (*M* = 4.68). Regarding perceived relief caused due to the use of the preferred application, the pathological (*M* = 4.49) and risky (*M* = 4.21) use groups indicated higher values than the non-problematic (*M* = 3.22) use group. For the strength of neglect of other activities due to the preferred application, there is again an incremental increase with a more problematic classification of symptom severity for PUI (*M*_np_ = 1.43; *M*_ri_ = 2.33; *M*_pa_ = 3.14; see Table 2).

### Multilevel analysis

#### Prediction of daily usage (RQ1).

To predict daily use of the respective online activity (yes/no) based on the severity of PUI symptoms, mood, stress, and temptation, the GLMM with random intercepts and cross-level interactions best fitted our data (*AIC* = 8714.0, *BIC* = 8809.1, *R²*_conditional_ = 79.9%, *R²*_marginal_ = 22.6%), compared to the random slopes model which did not converge (due to over-complexity) and the random intercepts model without cross-level interactions (∆*AIC*=+34.8, ∆*BIC* = −9.0, ∆*R²*_conditional_ = −0.1%, ∆*R²*_marginal_ = −0.1%). The random effects of the within clusters result in an approximated *ICC* of 73.99% (random effects = 9.358, *SD* = 3.059), which indicates that there is a high variance in the within clusters and a multilevel model should be used. Overall, 79.9% of the variance of daily usage (yes/no) can be explained through the model with all (random and fixed) effects and fixed effects alone explain 22.6% of the model variance. It is important to note that the conditional *R²* reflects within-person, between-person, as well as random effects and should not be interpreted as a purely cross-sectional predictive performance metric, for which the marginal *R²* is more appropriate. We also tested for collinearity and found that all *VIF*s are smaller than 4.0, indicating that there is only a low collinearity between all variables.

**Fig 2:**
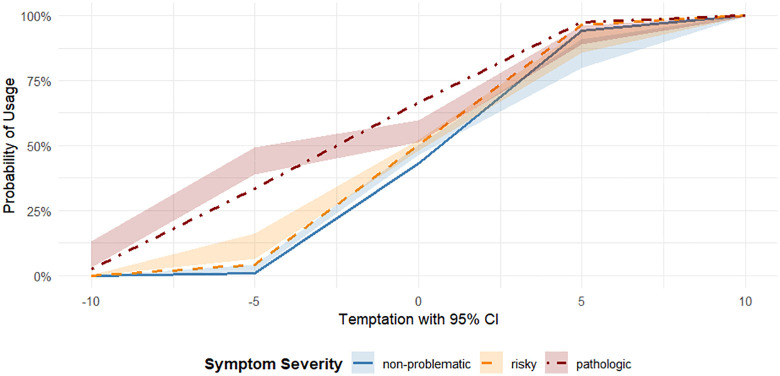
Probability of usage predicted by temptation and classification of symptom severity. Temptation is centered within clusters. The colored areas are the confidence intervals.

**Fig 3:**
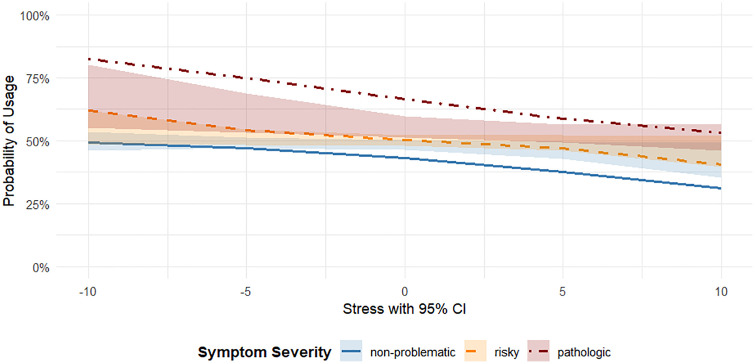
Probability of usage predicted by mood and symptom severity. Stress is centered within clusters. The colored areas are the confidence intervals.

**Fig 4:**
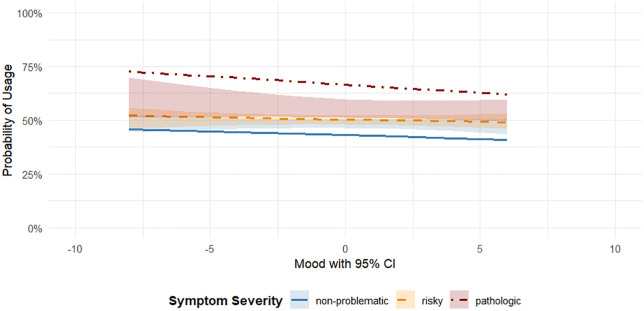
Probability of usage predicted by mood and symptom severity. Temptation is centered within clusters. The colored areas are the confidence intervals.

The baseline for the probability of use in the non-problematic use group is 23.15% lower than the baseline for the risky use group, which is again 30.12% lower than that of the pathological use group (see Table 3). In addition to symptom severity, temptation (*b* = 0.93, *p* < .001) is a strong positive predictor, stress (*b* = −0.07, *p* = .007) is a weak negative predictor, and mood (*b* = −0.03, *p* = .405) does not predict the probability of usage. The effect of temptation weakens with higher symptom severity (*b* = 0.61, *p* < .001) although the usage is overall higher with more symptom severity; all other cross-level interactions are not significant (see Table 3 and Figs 2–4). Given that the cross-level interactions with the risky and pathological group only provide information on whether the predictors show stronger/weaker effects with increasing symptom severity, the baseline effects nevertheless apply to all groups. Accordingly, when individuals experience more temptation than usual, the probability of usage increases, and when individuals experience more stress than usual, the probability of usage decreases slightly.

**Table 3:** Fixed effects of PUI symptom severity, mood, stress, temptation in the GLMM for the prediction of usage (yes/no).

Fixed Effects	*b*	*SE*	*z*	*p*	*OR*	Probability of usage	b adjusted for group
Intercept (NP)	−0.90	0.16	−5.48	<.001	0.41	28.85%	
RI	0.98	0.27	3.68	<.001	2.67	52.00%	
PA	2.43	0.28	8.70	<.001	11.33	82.12%	
Mood (NP)	−0.03	0.03	−0.83	.405	0.97		
Stress (NP)	−0.07	0.03	−2.70	.007	0.93		
Temptation (NP)	0.93	0.04	25.52	<.001	2.53		
RI*Mood	−0.02	0.05	−0.34	.733	0.98		−0.05
RI*Stress	−0.04	0.04	−0.97	.334	0.96		−0.12
RI*Temptation	−0.10	0.05	−1.93	.054	0.91		0.83
PA*Mood	−0.02	0.05	−0.32	.750	0.98		−0.04
PA*Stress	−0.02	0.04	−0.51	.609	0.98		−0.10
PA*Temptation	−0.32	0.05	−6.56	<.001	0.73		0.61

Note. NP = non-problematic group, RI = risky group, PA = pathologic group. Mood, stress and temptation are centered within clusters.

#### Prediction of pleasure, relief, and neglect of other activities on days with usage (RQ2).

To investigate the relations between mood, stress, temptation, use time, pleasure, relief and neglect, we calculated MG-ML-SEMs. For the dependent variables of pleasure, relief, and neglect, the *ICCs* are .466, .522, and .417, respectively, which indicates clear within-cluster effects, hence a multilevel model should be calculated.

**Fig 5:**
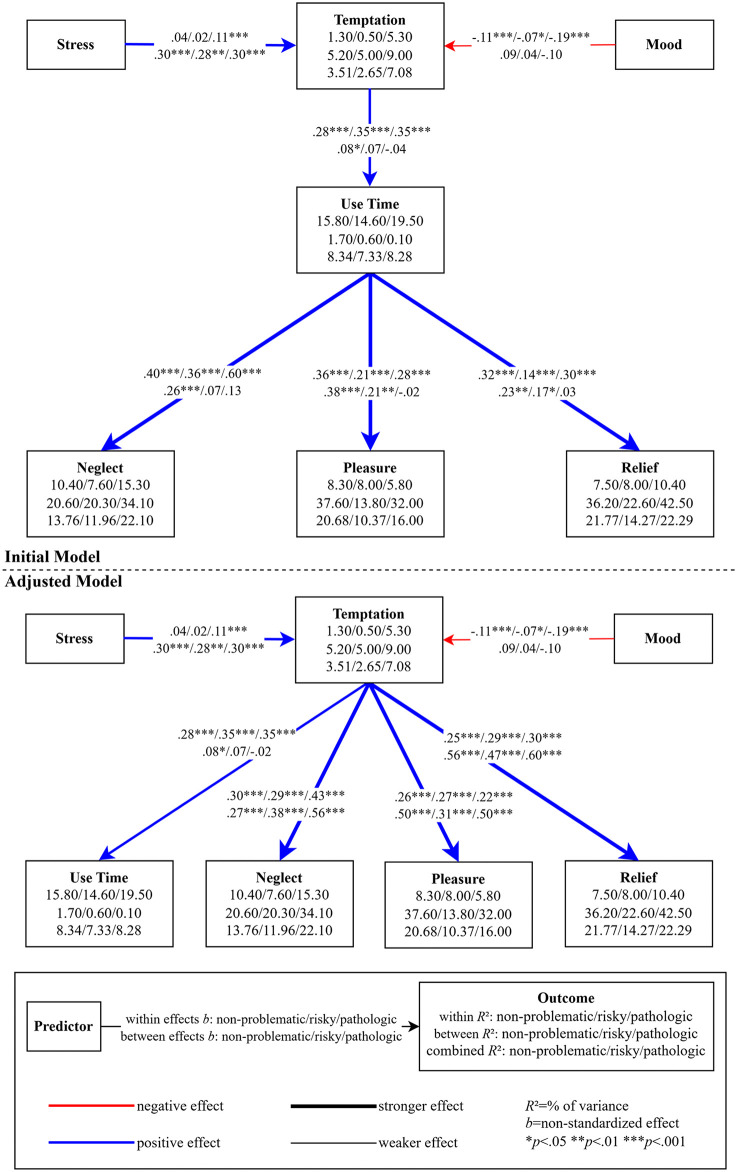
Initial model and use time adjustment: non-standardized effects of the multigroup multilevel structural equation model.

At first, we calculated our initial model (see Fig 1) including within- and between-person effects, which showed poor fit (see Table 4). This has led us to rethink the role of use time in the context of PUI symptom severity. Although use time is often used as an indicator of symptom severity and there are indeed empirically proven correlations, a high use time does not necessarily correspond to a high level of impairment or neglect of other important things, and vice versa [82–85]. And certainly, a high use time does not necessarily generate more pleasure and relief. Even a short session could trigger an intense feeling of pleasure and relief. To counteract the overpathologization of normal behavior involving high use time, we examined the modifier indices for use time. There are high covariances (*MI* = 13–104, see S7 Table) between use time and the other dependent variables, and additional paths from the dependent variables to use time are suggested (*MI* = 20–127, see S7 Table). Further, we saw no between-person effects of use time on neglect for the risky and pathologic group (see Fig 5 and S9 Table). Therefore, we decided to switch use time to a dependent variable. This decision was driven by theory and supported by the data.

**Table 4:** Fit indices of the multigroup multilevel structural equation models’ increments.

Initial Model	Non-problematic use group	Risky use group	Pathological use group
CFI	0.69	0.65	0.77
TLI	0.44	0.37	0.59
RMSEA	0.12	0.12	0.12
SABIC	40924.66	31276.58	38590.84
AIC	40838.58	31202.15	38508.68
BIC	41035.85	31387.77	38702.04
Adjusted Model			
CFI	0.87	0.82	0.92
TLI	0.68	0.54	0.79
RMSEA	0.09	0.10	0.08
SABIC	40419.18	30860.32	38150.23
AIC	40320.82	30775.26	38277.31
BIC	40546.27	30987.39	38695.17
Final (fully saturated) Model			
SABIC	40216.39	30652.55	38012.29
AIC	40078.68	30533.46	37880.82
BIC	40394.31	30830.44	38190.20

Note. CFI = Comparative Fit Index, TLI = Tucker-Lewis Index, RMSEA = Root Mean Square Error of Approximation, SABIC = Sample-size Adjusted Bayesian Information Criterion, AIC = Akaike’s Information Criterion, BIC = Bayesian Information Criterion.

**Fig 6:**
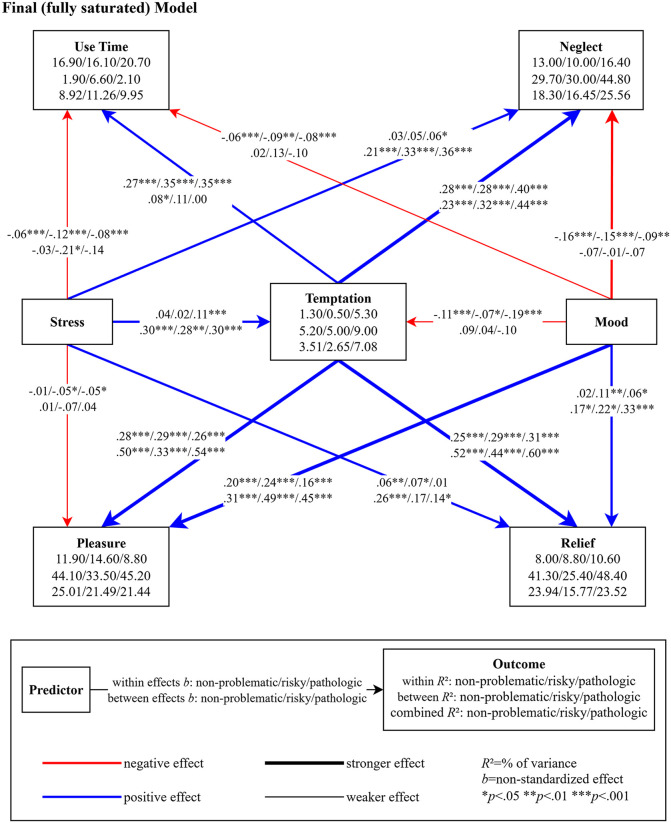
Final model: non-standardized effects of the multigroup multilevel structural equation model.

After this change, the fit indices improved considerably (see Table 4) but were still not optimal. Further considerations in the context of the current interpretations of the I-PACE model [9] led to the understanding that stress and mood exert their effects on the dependent variables via temptation as mediator; consequently, stress and mood should also have direct effects on perceived pleasure and relief, as well as on usage time and on neglect of other activities. This had previously been observed in other ambulatory assessment studies [43,44,53,54,60]. Furthermore, our additional analysis revealed that the modifier indices support this assumption. Consequently, we have added the additional relationship between stress and mood to the existing independent variables. However, this results in a completely saturated model, which is why CFI, TLI, and RMSEA can no longer be used as fit indices. Nevertheless, we can deduct from the SABIC, AIC, and BIC that the model has improved again and fits well (see Table 4). The use of saturated models increases the risk of overfitting. However, this is unlikely in the context of the present analyses due to the following factors: 1. All estimated parameters are theoretically motivated and were specified a-priori based on theory and prior empirical work while adhering to the principle of parsimony; 2. The standard errors of the parameters are comparatively small, which indicates high parameter stability; 3. The sample size is adequate for the numbers of parameters; 4. The key parameter estimates were robust across the previous calculated alternative, more parsimonious model specifications; and 5. The fit indices BIC/SABIC tend to be more sensitive to more complex models, thus lower BIC/SABIC values for the saturated model indicate a better fit compared to the more parsimonious models. Nevertheless, caution is advised when interpreting the results of fully saturated models in absolute terms, as the options for evaluating the model fit using additional standard fit indices – other than AIC and BIC – are limited. However, even though we have modified the position of one variable compared to the initial model and allowed for additional relationships between the already included variables, the direction and intensity of the effects remain largely unchanged. In fact, the assumed theoretical relationships were already confirmed in the first MG-ML-SEM, and the additional relationships actually allowed us to gain even deeper insights. For comparison of the effects, see Figs 5 and 6, as well as Tables 5, S9 and S10.

**Table 5:** Multigroup multilevel structure equation model: regression effects of the final saturated model.

Outcome	Predictor	Non-problematic use group					Risky use group					Pathological use group				
Level 1 (within-person)		*b*	*SE*	*z*	*p*	*β*	*b*	*SE*	*z*	*p*	*β*	*b*	*SE*	*z*	*p*	*β*
Temptation	Stress	0.04	0.02	1.81	0.070	0.04	0.02	0.03	0.57	0.569	0.02	0.11	0.02	4.50	<.001	0.11
	Mood	−0.11	0.03	−3.97	<.001	−0.09	−0.07	0.03	−2.22	0.027	−0.06	−0.19	0.03	−7.03	<.001	−0.17
Use Time	Temptation	0.27	0.01	19.68	<.001	0.40	0.35	0.02	16.03	<.001	0.38	0.35	0.02	20.74	<.001	0.44
	Stress	−0.06	0.01	−4.58	<.001	−0.10	−0.12	0.02	−5.10	<.001	−0.14	−0.08	0.02	−4.49	<.001	−0.10
	Mood	−0.06	0.02	−3.53	<.001	−0.08	−0.09	0.03	−3.19	0.001	−0.09	−0.08	0.02	−3.88	<.001	−0.09
Neglect	Temptation	0.28	0.02	14.79	<.001	0.31	0.28	0.03	10.75	<.001	0.27	0.40	0.02	16.79	<.001	0.37
	Stress	0.03	0.02	1.50	0.133	0.03	0.05	0.03	1.86	0.063	0.05	0.06	0.03	2.30	0.021	0.05
	Mood	−0.16	0.02	−6.84	<.001	−0.15	−0.15	0.03	−4.50	<.001	−0.12	−0.09	0.03	−3.40	0.001	−0.08
Pleasure	Temptation	0.28	0.02	14.90	<.001	0.31	0.29	0.02	12.58	<.001	0.30	0.26	0.02	12.32	<.001	0.28
	Stress	−0.01	0.02	−0.33	0.743	−0.01	−0.05	0.03	−2.02	0.043	−0.05	−0.05	0.02	−2.24	0.025	−0.05
	Mood	0.20	0.02	8.61	<.001	0.19	0.24	0.03	8.46	<.001	0.23	0.16	0.02	6.36	<.001	0.15
Relief	Temptation	0.25	0.02	12.80	<.001	0.27	0.29	0.03	11.53	<.001	0.29	0.31	0.02	14.68	<.001	0.33
	Mood	0.06	0.02	3.16	0.002	0.07	0.07	0.03	2.38	0.017	0.07	0.01	0.02	0.43	0.671	0.01
	Stress	0.02	0.02	0.74	0.463	0.02	0.11	0.03	3.46	0.001	0.10	0.06	0.03	2.32	0.020	0.06
Level 2 (between-person)		*b*	*SE*	*z*	*p*	*β*	*b*	*SE*	*z*	*p*	*β*	*b*	*SE*	*z*	*p*	*β*
Temptation	Stress	0.30	0.07	4.27	<.001	0.24	0.28	0.08	3.35	0.001	0.23	0.30	0.08	3.95	<.001	0.27
	Mood	0.09	0.08	1.13	0.259	0.06	0.04	0.10	0.36	0.716	0.03	−0.10	0.09	−1.11	0.267	−0.08
Use Time	Temptation	0.08	0.04	2.17	0.030	0.14	0.11	0.07	1.65	0.098	0.12	0.00	0.06	0.05	0.960	0.00
	Stress	−0.03	0.05	−0.60	0.550	−0.04	−0.21	0.09	−2.51	0.012	−0.20	−0.14	0.08	−1.88	0.060	−0.15
	Mood	0.02	0.06	0.30	0.763	0.02	0.13	0.10	1.36	0.175	0.10	−0.10	0.09	−1.12	0.263	−0.09
Neglect	Temptation	0.23	0.04	6.42	<.001	0.39	0.32	0.06	5.41	<.001	0.38	0.44	0.06	7.29	<.001	0.46
	Stress	0.21	0.05	4.37	<.001	0.28	0.33	0.08	4.33	<.001	0.32	0.36	0.07	5.02	<.001	0.34
	Mood	−0.07	0.06	−1.21	0.227	−0.08	−0.01	0.09	−0.06	0.956	0.00	−0.07	0.08	−0.86	0.389	−0.06
Pleasure	Temptation	0.50	0.04	11.95	<.001	0.61	0.33	0.06	6.08	<.001	0.40	0.54	0.06	9.83	<.001	0.61
	Stress	0.01	0.06	0.20	0.839	0.01	−0.07	0.07	−1.02	0.306	−0.07	0.04	0.07	0.65	0.519	0.04
	Mood	0.31	0.06	4.93	<.001	0.26	0.49	0.08	6.16	<.001	0.42	0.45	0.07	6.16	<.001	0.39
Relief	Temptation	0.52	0.05	10.98	<.001	0.55	0.44	0.07	6.65	<.001	0.45	0.60	0.06	10.73	<.001	0.65
	Mood	0.26	0.06	4.26	<.001	0.23	0.17	0.09	1.93	0.053	0.14	0.14	0.07	2.15	0.032	0.14
	Stress	0.17	0.07	2.38	0.017	0.12	0.22	0.10	2.23	0.026	0.16	0.33	0.08	4.36	<.001	0.27

Note. The within-person predictor variables are centered within cluster. The between-person predictor variables are grand mean centered.

Stress mainly appears as a between factor. The results (see Table 5; Level 2) indicate that people with overall higher stress levels experience more temptation (*b*’s from .28 to .30), more neglect (*b*’s from .21 to .36), more relief (*b*’s from .17 to .33) with the effects of neglect and relief being amplified by the severity of PUI symptoms. Within effects show that use time decreases on stressful days (*b*’s from −.12 to −.06), but less for high PUI symptom severity. Congruently – only for the pathological users – temptation (*b* = .11) and neglect (*b* = .06) increase with higher stress. The effects of stress over temptation show for pathological users, that although the use time decreases on stressful days, this group still engages in the online activity which leads to more neglect. Furthermore, on days with higher perceived stress, individuals with risky and pathological use experience slightly less pleasure (*b*’s = −.05) and slightly more relief (*b*’s from .06 to .11) through the activity (see Table 5 and Fig 6).

Mood showed bidirectional relationships. On days with a worse mood (within-person effects; see Table 5, Level 1), temptation (*b*’s from −.19 to −.07), use time (*b*’s from −.09 to −.06) and neglect (*b*’s from −.16 to −.09) increase, while experienced pleasure (*b*’s from .26 to .29) and relief (*b*’s from .06 to .07) decrease. Concerning the between-person effects (see Table 5; Level 2), people with a better mood overall experience more pleasure (*b* = .33–.50, strongest for risky and pathological use) and relief (*b* = .14–.26, strongest for non-problematic use) through the activity. An improved overall mood also predicts more pleasure in everyday life. Although a worse mood leads to more usage, the bad mood is not improved (compensated) by the use itself, especially for people with high symptom severity of PUI (see Table 5 and Fig 6). We saw no significant between-person effects of mood on temptation, neglect and use time, which supports the results from the GLMM, that mood is not a central predictor of actual use of the Internet.

Temptation to use seems to be the main driving factor. The within and between-person effects of temptation on pleasure, relief, and neglect are moderate to strong and positive (*b*’s from .23 to .60; see Table 5). An average higher temptation between persons and days with increased temptation (within), result in more pleasure, relief and neglect. Thus, temptation is a strong predictor in every respect. Viewed by group, the pathological use group has the strongest effects between temptation and neglect. For all groups, the use time is higher on days with more temptation (strong within-person effects). In contrast the between-person effects are minimal to non-existent (*b*’s < .08), indicating that a higher average temptation does not necessarily correspond to higher average use times (see Table 5 and Fig 6), which supports again the assumption that a general high use time does not necessarily equate to the PUI symptom severity.

## Discussion

With this study we analyzed a 14-day ambulatory end-of-day assessment in individuals with non-problematic, risky, or pathological Internet use across different types of PUI. Concerning research question 1a, the results of our multigroup multilevel analyses indicate that daily perceived strength of temptation to use is the main predictor for engaging in a (problematic) online activity for all groups (non-problematic, risky and pathological) on that day, increased temptation leads to an increased probability to use the Internet. This is consistent with the existing literature, which identifies craving as a key predictor in cross-sectional and daily assessments [48,50,54,56]. The finding is also in accordance with the I-PACE model, although temptation is not conceptually equivalent to craving [7]. Craving is typically defined as a more intense and sometimes persistent urge with affective and cognitive elaboration, especially in the context of addiction [86,87]. Temptation however, integrates several hedonic components commonly associated with craving, including desire, salience, and perceived difficulty resisting engagement [88,89], which can also be experienced by individuals with low symptom severity. Accordingly, in the present study, “temptation to use” is used as a pragmatic proxy for craving-related motivational processes rather than as a comprehensive measure of craving. Besides that, we found a small negative effect of perceived daily stress on the probability of use (more stress = lower probability of use) and no effect of daily mood, which is similar to the findings of other studies [36,43,90]. As stress and relief are normally distributed and show no anomalies concerning skewness and kurtosis, we also assume a floor effect to be unlikely, but the results are mixed and literature is scarce. The small effects of stress and mood for the prediction of usage could suggest, that these affective states may not be direct triggers for behavior enactment, but they may rather be related to PUI in the sense of a worsened well-being (irrespective of usage).

Results of group comparisons at baseline (research question 1b) show that the probability of use in the following 14 days increases most strongly in the pathological group, less strongly in the risky group and least in the non-problematic group. Further, people with pathological Internet use show worse mood, more daily stress, and higher temptation, compared to people with a non-problematic use, which is in line with the previous (cross-sectional) findings [31,42,49,53] and models of PUI [7].

Concerning research question 2a, the fully saturated model (and therefore possibly not an ideal model) shows that, more stress in daily life leads to higher temptation to use as well as to more experienced relief through the online activity and to more neglect of other activities on a daily basis over 14 days. These effects are stronger with a higher PUI symptom severity (research question 2b). Similar connections have been found in other daily assessments [37,54] and this specific connection was addressed theoretically in the I-PACE model [7]. Days with more stress are predictive of decreased use time, probably due to time constraints. This effect is weaker for individuals with pathological PUI and is accompanied by more temptation and neglect of other activities. This might be indicative of the online activities being continued to be pursued despite an increased/predominant workload, which reflects continuation of the behavior despite the experience of negative consequences, which is a diagnostic criterion for addictive behaviors [2]. Also interesting is the finding that for individuals with risky and pathological Internet use, higher daily stress predicts more experienced relief but less experienced pleasure through the online activity. In general, people with a pathological Internet use experience more stress in daily life, which was also shown before [21,32,33] and is in line with the current interpretation of the I-PACE model [9].

Mood shows some similar effects to stress. On days with a worse mood, negative effects are amplified: higher temptation, longer use times, and more neglect is reported, which corresponds to common models of behavioral addictions [7,8]. Better mood is predictive of positive effects, namely, experienced relief and pleasure are increased. So, bad mood may lead to an intensification of a (problematic) online activity but also allows less gratifying and compensating feelings. Daily mood in general seems to be worse with more severe PUI, potentially reflecting impairments in daily life. Similar effects have been found in a 7-day ecological momentary assessment, where social media detox led to a reduction of negative affect and boredom, but also to a reduction of positive affect in general [91]. Which shows again, similar to stress, that mood may not be a direct trigger for a behavior enactment, but has – in combination with PUI – impacts on the general well-being and shows reinforcing effect concerning pleasure and relief potentially leading to an accelerated development of PUI.

Temptation is the main predictor for all other daily outcomes of PUI, even in the more complex model. On average, on days with higher temptation, more time is spent online, more pleasure and more relief are experienced, and more neglect occurs, which is again in line with the literature [50,54,56]. Especially the negative effects are amplified for more severe PUI (more severe PUI classification = higher use times and more neglect). People with increased temptation overall report more neglect, pleasure and relief through the online activities but do not indicate longer use times.

Additionally, our results show that experienced pleasure is the highest for the risky group and relevant for all stages of addiction and that relief increases with more PUI symptoms, which coincided with the current interpretations of the I-PACE model, in which it is stated that with rising symptom severity of PUI, more compensation is experienced, whereas gratification might be especially important for the initial development of addiction and remains important throughout the course of addiction [9]. People with more symptoms overall also show longer use times and more neglect accompanied with more stress and a worsened mood, which is consistent with findings of daily assessment studies, e.g., for gaming [92].

Against our initial assumptions, we found empirically, that use time turned out to better fit as an outcome variable than as a mediator. Thus, a high use time does not necessarily reflect high impairment [82]. This is also reflected in other daily assessment studies where only a weak relationship between behavior enactment and symptom severity was found [93,94]. A further nuance is that less relief is experienced with bad mood and higher symptom severity, which presumably causes increased use and may be due to behavior automatization [26]. We also found that mood and stress are weak predictors for the probability of daily use, which could indicate that these are not direct triggers for the usage but could be more relevant for some individuals than for others and may have more indirect effects on the usage over temptation [26,95].

Overall, it can be summarized that higher perceived stress in daily life predicts less use time but creates higher temptation and, especially in the pathological group, leads to more neglect of other activities. Higher stress further predicts that more relief and slightly less pleasure are derived from the behavior. A positive mood appears to have a protective effect, while a negative mood may result in heightened temptation and longer use times, with the negative mood becoming more difficult to compensate for as the severity of PUI symptoms increases. Previous temptation is predictive of feeling more pleasure and relief due to the behavior, but also leads to more neglect with increased use times. With more PUI symptom severity these effects are amplified.

### Limitations and recommendations

Although the conducted study follows current recommendations for ambulatory assessments (see [18]) and was designed strictly according to theoretical and empirically based assumptions, there are limitations that should be considered. First, due to economic reasons, the daily assessment was an end-of-day assessment. Thus, only one timepoint per day was assessed, which makes it difficult to determine in which order stress, pleasure, usage, neglect etc. occurred. A more finely grained assessment would be useful, but the timepoints should be similar for each participant, to have better within comparisons [18]. For the MG-ML-SEM, only active days were considered; thus, it is unclear whether pleasure, relief, or neglect were experienced on days without usage. Future studies should also address potential (other) outcomes of PUI on inactive days. The sample in this study mainly consist of young adults, which is typical for PUI, but limits the generalizability of the findings to the general population. We also used a fully saturated model, which limits interpretability and may result in overfitting. While the effects observed in the less complex models are similar, further research should nevertheless examine the mediating role of temptation via affective processes on internet use and its outcomes. Further, in contrast to the extensive diagnostic interview for the PUI symptom severity, we only included single items for the daily assessments (also for ecological reasons). There are certainly more facets to each construct than we assessed, e.g., gratification has the facets of *gratification of needs* and *experience of pleasure* or compensation has the facets of *compensation of needs* and *experience of relief from negative feelings* [11,59] and craving has in addition to temptation/desire, the facets of urges and habitually driven craving [96], but we had to condense these facets in order to test the entire model and avoid having to compromise on the number of assessed constructs. In addition to the assessed constructs, compulsivity and impaired control could also be addressed in future studies. The inclusion of more facets could bring more in-depth insights. This could be achieved with smaller studies assessing each construct in detail. In addition, a more detailed analysis of specific PUI subtypes could reveal further insights into similarities and differences between PUI types (gaming, buying-shopping, social network use, pornography use, etc.). Although common mechanisms are assumed and analyzed, PUI subtype-specific usage characteristics may also be present. This could be different motivations and triggers to use an application (e.g., sexual desire for pornography use). Future studies that aim to identify subtype-specific mechanisms may use a similar approach but focusing on more specific usage types. Also, the inclusion of more objective measurements could be beneficial, as self-report data of, e.g., use time, stress or pleasure might differ from actual use times, or biochemical/physiological indicators of stress [97,98].

## Conclusion

With this 14-day end-of-day ambulatory assessment study we could identify connections between daily stress and mood over temptation to engage in an online activity (gaming, buying-shopping, pornography use, social network use) and their prediction of the experience of pleasure and relief, as well as increased use time and neglect of other obligations due to the online activity across different types of PUI and between different degrees of PUI severity. Whereas daily temptation is the main predictor for the probability of engaging in an online activity and for predicting the other related outcomes. On days with more temptation, more time is spent online, more pleasure and more relief are experienced and more neglect occurs. With more PUI symptom severity, overall more stressful days and a worse daily mood are reported which is in accordance with etiological models on the development and maintenance of addictive behaviors [7]. Looking at the effects separately for different PUI severity groups, it shows that over the course of addiction development, the daily mood may decrease gradually, the temptation increases gradually, whereas perceived stress mainly increases in later stages of addiction. More daily stress leads to higher temptations, more relief through the online activity and to more neglect, meaning especially negative effects are amplified with more severe PUI. This applies also to daily mood. With more PUI severity, the decreased daily mood leads to an intensification of a behavior as less gratifying and compensating feelings are experienced and the online activity must be engaged in more frequently. We can conclude that in daily life 1. more stress, worse mood and higher temptation to use play an important role in the engagement with online activities, and that 2. throughout the development of PUI, experienced pleasure (gratification) and relief (compensation) are reinforcing factors and 3. that with a more severe PUI, negative effects are intensified, i.e., more neglect and longer use times (interferences), more stress and worse mood (marked distress) are experienced in daily life. The role of stress and mood as direct triggers for internet use remains to be clarified. Against this backdrop, intervention strategies could focus on identifying increased cravings and offer functional alternatives that help provide pleasure and relief without leading to the neglect of other important aspects of life (e.g., work or relationships).

With the extensive sample collected in this study and the robust statistical methods used we were able to show that assumptions about the interplay of affective and cognitive processes in common theoretical models of PUI can also be found in everyday life and differ in their effects depending on PUI severity. The insights into affective mechanisms, reinforcing effects, and impairments are transferable to potential therapeutic approaches. Based on the findings of the current study, interventions addressing PUI should focus in particular on the emergence and regulation of temptations to use the Internet in everyday life.

## Supporting information

S1 Table Gender distribution of the sample. (DOCX)

S2 Table School education (German system) distribution of the sample. (DOCX)

S3 Table Professional education degree distribution of the sample. (DOCX)

S4 Table Employment distribution of the sample. (DOCX)

S5 Table Marital status distribution of the sample. (DOCX)

S6 Table Partnership distribution of the sample. (DOCX)

S7 Table Modifier Indices>10 and (standardized) expectations of parameter change for theoretical model. (DOCX)

S8 Table Modifier Indices>10 and (standardized) expectations of parameter change for model with adjustment of use time. (DOCX)

S9 Table Multigroup multilevel structure equation model: regression effects of the theoretical model. (DOCX)

S10 Table Multigroup multilevel structure equation model: regression effects of the model with adjustment of use time. (DOCX)

S11 Dataset Anonymized dataset for this study. (SAV)
