# Supplementary material for: Temptation as a key driver between affective states and usage outcomes of problematic usage of the Internet: A 14-day ambulatory assessment study
Source: PLoS One. 2026 Jul 29;21(7):e0352776. doi: 10.1371/journal.pone.0352776 (PMC13419235; doi:10.1371/journal.pone.0352776)
Supplement: S1 Table — (DOCX) [file pone.0352776.s001.docx]

| **Table S1. Gender distribution of the sample.** | | |
| --- | --- | --- |
| Gender | Amount | % |
| Female | 413 | 45.89 |
| Male | 482 | 53.56 |
| Other | 5 | 0.55 |
