# Supplementary material for: Temptation as a key driver between affective states and usage outcomes of problematic usage of the Internet: A 14-day ambulatory assessment study
Source: PLoS One. 2026 Jul 29;21(7):e0352776. doi: 10.1371/journal.pone.0352776 (PMC13419235; doi:10.1371/journal.pone.0352776)
Supplement: S2 Table — (DOCX) [file pone.0352776.s002.docx]

| **Table S2. School education (German system) distribution of the sample.** | | |
| --- | --- | --- |
| School/academic education | Amount | % |
| Secondary school certificate (Hauptschulabschluss) | 11 | 1.22 |
| Intermediate school leaving certificate (Realschulabschluss) | 54 | 6.00 |
| Advanced technical college entrance qualification (Fachabitur) | 84 | 9.33 |
| General higher education entrance qualification (Abitur) | 742 | 82.44 |
| Still a pupil | 6 | 0.67 |
| Other | 3 | 0.33 |
