# Supplementary material for: Temptation as a key driver between affective states and usage outcomes of problematic usage of the Internet: A 14-day ambulatory assessment study
Source: PLoS One. 2026 Jul 29;21(7):e0352776. doi: 10.1371/journal.pone.0352776 (PMC13419235; doi:10.1371/journal.pone.0352776)
Supplement: S3 Table — (DOCX) [file pone.0352776.s003.docx]

| **Table S3. Professional education degree distribution of the sample.** | | |
| --- | --- | --- |
| Professional education degree | Amount | % |
| Completion of vocational-school education (vocational or commercial school) | 106 | 11.78 |
| Completion of vocational in-company training (apprenticeship) | 31 | 3.44 |
| Graduation from a technical school, master craftsman or technician school, vocational or technical academy | 18 | 2.00 |
| Polytechnic degree | 34 | 3.78 |
| University degree | 239 | 26.56 |
| No educational qualification | 49 | 5.44 |
| Still in training / studying / a pupil | 415 | 46.11 |
| Other | 8 | 0.89 |
