# Supplementary material for: Temptation as a key driver between affective states and usage outcomes of problematic usage of the Internet: A 14-day ambulatory assessment study
Source: PLoS One. 2026 Jul 29;21(7):e0352776. doi: 10.1371/journal.pone.0352776 (PMC13419235; doi:10.1371/journal.pone.0352776)
Supplement: S4 Table — (DOCX) [file pone.0352776.s004.docx]

| **Table S4. Employment distribution of the sample.** | | |
| --- | --- | --- |
| Employment | Amount | % |
| Pupil | 10 | 1.11 |
| Student | 625 | 69.44 |
| Voluntary service | 5 | 0.56 |
| Trainee/apprentice/retrainee | 18 | 2.00 |
| Housewife/househusband (family work) | 6 | 0.67 |
| Retired person/retiree, in early retirement | 3 | 0.33 |
| Not gainfully employed for other reasons | 12 | 1.33 |
| Part-time employed | 63 | 7.00 |
| Full-time employed | 121 | 13.44 |
| Other | 37 | 4.11 |
