# Supplementary material for: Temptation as a key driver between affective states and usage outcomes of problematic usage of the Internet: A 14-day ambulatory assessment study
Source: PLoS One. 2026 Jul 29;21(7):e0352776. doi: 10.1371/journal.pone.0352776 (PMC13419235; doi:10.1371/journal.pone.0352776)
Supplement: S5 Table — (DOCX) [file pone.0352776.s005.docx]

| **Table S5.** **Marital status distribution of the sample.** | | |
| --- | --- | --- |
| Marital status | Amount | % |
| Married/ registered civil partnership | 75 | 8.33 |
| Divorced | 14 | 1.56 |
| Widowed | 1 | 0.11 |
| Single | 798 | 88.67 |
| Other | 12 | 1.33 |
