# Supplementary material for: Temptation as a key driver between affective states and usage outcomes of problematic usage of the Internet: A 14-day ambulatory assessment study
Source: PLoS One. 2026 Jul 29;21(7):e0352776. doi: 10.1371/journal.pone.0352776 (PMC13419235; doi:10.1371/journal.pone.0352776)
Supplement: S6 Table — (DOCX) [file pone.0352776.s006.docx]

| **Table S6. Partnership distribution of the sample.** | | |
| --- | --- | --- |
| Partnership | Amount | % |
| Yes | 504 | 56.00 |
| No | 390 | 43.33 |
| other | 6 | 0.67 |
