# Supplementary material for: Temptation as a key driver between affective states and usage outcomes of problematic usage of the Internet: A 14-day ambulatory assessment study
Source: PLoS One. 2026 Jul 29;21(7):e0352776. doi: 10.1371/journal.pone.0352776 (PMC13419235; doi:10.1371/journal.pone.0352776)
Supplement: S7 Table — (DOCX) [file pone.0352776.s007.docx]

| **Table S7.** **Modifier Indices>10 and (standardized) expectations of parameter change for the initial model.** | | | | | | |
| --- | --- | --- | --- | --- | --- | --- |
| Non-problematic use group | | | | | | |
| Outcome | Relation | Predictor | Level | MI | EPC | SEPC |
| Use Time* | Regression | Neglect | 1 | 127 | -0.40 | -0.58 |
| Temptation | Regression | Pleasure | 1 | 122 | 0.32 | 0.29 |
| Use Time* | Covariance | Neglect | 1 | 104 | -0.87 | -0.55 |
| Neglect | Regression | Temptation | 1 | 104 | 0.23 | 0.25 |
| Temptation | Regression | Neglect | 1 | 104 | 0.29 | 0.27 |
| Pleasure | Regression | Mood | 1 | 103 | 0.22 | 0.21 |
| Use Time* | Regression | Pleasure | 1 | 101 | -0.36 | -0.52 |
| Use Time* | Regression | Relief | 1 | 97 | -0.36 | -0.51 |
| Use Time* | Regression | Relief | 2 | 97 | -3.03 | -4.49 |
| Use Time* | Regression | Pleasure | 2 | 96 | -3.69 | -4.74 |
| Temptation | Regression | Relief | 1 | 92 | 0.28 | 0.26 |
| Temptation | Regression | Pleasure | 2 | 88 | 0.73 | 0.59 |
| Temptation | Covariance | Neglect | 1 | 84 | 0.62 | 0.23 |
| Temptation | Regression | Relief | 2 | 78 | 0.56 | 0.52 |
| Neglect | Regression | Mood | 1 | 68 | -0.20 | -0.18 |
| Temptation | Covariance | Pleasure | 1 | 62 | 0.49 | 0.19 |
| Mood | Regression | Pleasure | 1 | 61 | 0.16 | 0.17 |
| Mood | Regression | Neglect | 1 | 57 | -0.16 | -0.17 |
| Use Time* | Regression | Neglect | 2 | 50 | -3.90 | -3.64 |
| Pleasure | Regression | Temptation | 1 | 44 | 0.14 | 0.15 |
| Use Time* | Covariance | Pleasure | 1 | 44 | -0.52 | -0.33 |
| Temptation | Regression | Neglect | 2 | 35 | 0.68 | 0.40 |
| Relief | Regression | Stress | 1 | 28 | 0.10 | 0.11 |
| Relief | Regression | Temptation | 1 | 28 | 0.11 | 0.12 |
| Use Time* | Covariance | Relief | 1 | 28 | -0.42 | -0.27 |
| Neglect | Regression | Stress | 1 | 28 | 0.10 | 0.12 |
| Stress | Regression | Use Time | 1 | 24 | -0.19 | -0.11 |
| Temptation | Covariance | Pleasure | 2 | 24 | 0.70 | 0.23 |
| Temptation | Covariance | Relief | 1 | 22 | 0.29 | 0.11 |
| Pleasure | Regression | Temptation | 2 | 20 | 0.16 | 0.20 |
| Use Time* | Covariance | Pleasure | 2 | 20 | -3.08 | -1.57 |
| Pleasure | Regression | Mood | 2 | 16 | 0.21 | 0.18 |
| Neglect | Regression | Stress | 2 | 16 | 0.17 | 0.24 |
| Relief | Regression | Stress | 2 | 16 | 0.19 | 0.17 |
| Use Time | Regression | Stress | 1 | 15 | -0.05 | -0.08 |
| Pleasure | Regression | Stress | 1 | 15 | -0.07 | -0.08 |
| Mood | Regression | Use Time | 1 | 15 | -0.12 | -0.09 |
| Relief | Regression | Mood | 1 | 15 | -0.08 | -0.08 |
| Stress | Regression | Relief | 1 | 13 | 0.09 | 0.08 |
| Use Time* | Covariance | Neglect | 2 | 13 | -2.38 | -1.66 |
| Neglect | Regression | Temptation | 2 | 13 | 0.12 | 0.21 |
| Risky use group | | | | | | |
| Outcome | Relation | Predictor | Level | MI | EPC | SEPC |
| Temptation | Regression | Relief | 1 | 114 | 0.32 | 0.32 |
| Use Time* | Regression | Relief | 1 | 111 | -0.55 | -0.66 |
| Temptation | Regression | Pleasure | 1 | 103 | 0.33 | 0.31 |
| Pleasure | Regression | Mood | 1 | 95 | 0.25 | 0.23 |
| Use Time* | Regression | Pleasure | 1 | 87 | -0.53 | -0.59 |
| Mood | Regression | Pleasure | 1 | 60 | 0.18 | 0.19 |
| Use Time* | Regression | Neglect | 1 | 55 | -0.38 | -0.48 |
| Temptation | Regression | Neglect | 1 | 47 | 0.20 | 0.22 |
| Use Time* | Covariance | Neglect | 1 | 46 | -1.13 | -0.46 |
| Neglect | Regression | Temptation | 1 | 46 | 0.21 | 0.20 |
| Pleasure | Regression | Stress | 1 | 44 | -0.15 | -0.16 |
| Use Time* | Regression | Relief | 2 | 41 | -6.61 | -6.67 |
| Temptation | Covariance | Neglect | 1 | 39 | 0.60 | 0.19 |
| Temptation | Covariance | Pleasure | 1 | 37 | 0.47 | 0.16 |
| Relief | Regression | Temptation | 1 | 37 | 0.16 | 0.16 |
| Use Time* | Covariance | Relief | 1 | 37 | -0.90 | -0.36 |
| Neglect | Regression | Mood | 1 | 37 | -0.19 | -0.16 |
| Temptation | Regression | Relief | 2 | 36 | 0.45 | 0.44 |
| Use Time* | Regression | Neglect | 2 | 35 | -7.15 | -6.25 |
| Temptation | Covariance | Relief | 1 | 35 | 0.49 | 0.16 |
| Pleasure | Regression | Mood | 2 | 32 | 0.39 | 0.34 |
| Neglect | Regression | Stress | 1 | 31 | 0.16 | 0.15 |
| Stress | Regression | Use Time | 1 | 30 | -0.16 | -0.14 |
| Temptation | Regression | Pleasure | 2 | 29 | 0.50 | 0.41 |
| Pleasure | Regression | Temptation | 1 | 28 | 0.13 | 0.14 |
| Use Time* | Covariance | Pleasure | 1 | 28 | -0.72 | -0.31 |
| Use Time* | Regression | Pleasure | 2 | 25 | -6.41 | -5.44 |
| Temptation | Regression | Neglect | 2 | 24 | 0.43 | 0.37 |
| Mood | Regression | Neglect | 1 | 24 | -0.10 | -0.12 |
| Neglect | Regression | Stress | 2 | 21 | 0.34 | 0.34 |
| Use Time | Regression | Stress | 1 | 19 | -0.09 | -0.10 |
| Relief | Regression | Stress | 1 | 19 | 0.11 | 0.10 |
| Neglect | Regression | Temptation | 2 | 19 | 0.27 | 0.32 |
| Use Time* | Covariance | Neglect | 2 | 19 | #### | -4.40 |
| Mood | Regression | Use Time | 1 | 12 | -0.09 | -0.09 |
| Temptation | Covariance | Neglect | 2 | 11 | 0.61 | 0.25 |
| Pathological use group | | | | | | |
| Outcome | Relation | Predictor | Level | MI | EPC | SEPC |
| Use Time* | Regression | Neglect | 1 | 126 | -0.40 | -0.58 |
| Temptation | Regression | Relief | 1 | 124 | 0.32 | 0.31 |
| Use Time* | Regression | Relief | 1 | 121 | -0.42 | -0.53 |
| Temptation | Regression | Neglect | 1 | 89 | 0.25 | 0.28 |
| Neglect | Regression | Temptation | 1 | 88 | 0.25 | 0.23 |
| Use Time* | Covariance | Neglect | 1 | 88 | -1.18 | -0.48 |
| Use Time* | Regression | Relief | 2 | 81 | 14.40 | 14.32 |
| Temptation | Regression | Pleasure | 1 | 77 | 0.26 | 0.24 |
| Temptation | Regression | Relief | 2 | 74 | 0.66 | 0.60 |
| Temptation | Regression | Pleasure | 2 | 64 | 0.64 | 0.56 |
| Use Time* | Regression | Neglect | 2 | 62 | 12.12 | 12.72 |
| Use Time* | Regression | Pleasure | 2 | 61 | 12.98 | 12.47 |
| Pleasure | Regression | Mood | 1 | 57 | 0.16 | 0.16 |
| Temptation | Covariance | Neglect | 1 | 57 | 0.73 | 0.20 |
| Relief | Regression | Temptation | 1 | 52 | 0.16 | 0.17 |
| Use Time* | Covariance | Relief | 1 | 52 | -0.74 | -0.32 |
| Use Time* | Regression | Pleasure | 1 | 49 | -0.27 | -0.33 |
| Neglect | Regression | Stress | 1 | 46 | 0.16 | 0.15 |
| Temptation | Covariance | Relief | 1 | 44 | 0.51 | 0.15 |
| Temptation | Regression | Neglect | 2 | 39 | 0.46 | 0.44 |
| Neglect | Regression | Mood | 1 | 36 | -0.16 | -0.13 |
| Neglect | Regression | Stress | 2 | 33 | 0.42 | 0.39 |
| Mood | Regression | Pleasure | 1 | 28 | 0.12 | 0.12 |
| Neglect | Regression | Temptation | 2 | 26 | 0.33 | 0.35 |
| Use Time* | Covariance | Neglect | 2 | 26 | 23.44 | 7.66 |
| Stress | Regression | Use Time | 1 | 23 | -0.16 | -0.12 |
| Mood | Regression | Neglect | 1 | 22 | -0.09 | -0.11 |
| Pleasure | Regression | Stress | 1 | 21 | -0.09 | -0.10 |
| Mood | Regression | Use Time | 1 | 17 | -0.12 | -0.10 |
| Neglect | Regression | Mood | 2 | 15 | -0.33 | -0.26 |
| Use Time | Regression | Stress | 1 | 13 | -0.06 | -0.08 |
| Temptation | Covariance | Pleasure | 1 | 12 | 0.27 | 0.08 |
| Pleasure | Regression | Mood | 2 | 12 | 0.20 | 0.17 |
| Temptation | Covariance | Neglect | 2 | 12 | 0.76 | 0.24 |
| Use Time* | Covariance | Relief | 2 | 11 | 10.18 | 3.48 |
| Relief | Regression | Temptation | 2 | 11 | 0.14 | 0.16 |
| *Relevant to the decision regarding model adjustments | | | | | | |
