# Supplementary material for: Temptation as a key driver between affective states and usage outcomes of problematic usage of the Internet: A 14-day ambulatory assessment study
Source: PLoS One. 2026 Jul 29;21(7):e0352776. doi: 10.1371/journal.pone.0352776 (PMC13419235; doi:10.1371/journal.pone.0352776)
Supplement: S8 Table — (DOCX) [file pone.0352776.s008.docx]

| **Table S8. Modifier Indices>10 and (standardized) expectations of parameter change for the adjusted model.** | | | | | | |
| --- | --- | --- | --- | --- | --- | --- |
| Non-problematic use group | | | | | | |
| Outcome | Relation | Predictor | Level | MI | EPC | SEPC |
| Pleasure* | Regression | Mood | 1 | 111 | 0.23 | 0.22 |
| Temptation | Covariance | Pleasure | 1 | 104 | 5.09 | 1.94 |
| Temptation | Regression | Pleasure | 1 | 82 | 2.14 | 1.96 |
| Mood | Regression | Pleasure | 1 | 80 | 0.20 | 0.21 |
| Temptation | Regression | Neglect | 1 | 65 | -1.89 | -1.75 |
| Temptation | Covariance | Neglect | 1 | 59 | -4.08 | -1.55 |
| Neglect* | Regression | Mood | 1 | 54 | -0.17 | -0.16 |
| Mood | Regression | Neglect | 1 | 50 | -0.15 | -0.16 |
| Relief* | Regression | Stress | 1 | 24 | 0.09 | 0.10 |
| Stress | Regression | Use Time | 1 | 24 | -0.18 | -0.12 |
| Temptation | Covariance | Relief | 1 | 22 | -2.34 | -0.89 |
| Use Time* | Regression | Stress | 1 | 20 | -0.06 | -0.09 |
| Neglect* | Regression | Stress | 1 | 20 | 0.08 | 0.10 |
| Pleasure* | Regression | Mood | 2 | 19 | 0.21 | 0.18 |
| Pleasure* | Regression | Stress | 1 | 18 | -0.08 | -0.09 |
| Temptation | Regression | Neglect | 2 | 18 | -2.31 | -1.37 |
| Temptation | Regression | Relief | 2 | 16 | -1.43 | -1.34 |
| Mood | Regression | Use Time | 1 | 14 | -0.11 | -0.09 |
| Neglect* | Regression | Stress | 2 | 13 | 0.16 | 0.21 |
| Relief* | Regression | Stress | 2 | 13 | 0.18 | 0.15 |
| Temptation | Covariance | Relief | 2 | 13 | -2.39 | -0.80 |
| Relief* | Regression | Mood | 1 | 12 | -0.08 | -0.07 |
| Stress* | Regression | Relief | 1 | 11 | 0.09 | 0.08 |
| Temptation | Covariance | Neglect | 2 | 11 | -1.92 | -0.91 |
| Risky use group | | | | | | |
| Outcome | Relation | Predictor | Level | MI | EPC | SEPC |
| Temptation | Regression | Pleasure | 1 | 118 | 4.50 | 4.27 |
| Temptation | Covariance | Pleasure | 1 | 110 | 10.04 | 3.52 |
| Pleasure* | Regression | Mood | 1 | 103 | 0.26 | 0.24 |
| Mood | Regression | Pleasure | 1 | 74 | 0.21 | 0.22 |
| Pleasure* | Regression | Stress | 1 | 49 | -0.16 | -0.16 |
| Temptation | Regression | Neglect | 1 | 42 | -2.38 | -2.53 |
| Temptation | Covariance | Neglect | 1 | 34 | -6.81 | -2.14 |
| Pleasure* | Regression | Mood | 2 | 33 | 0.39 | 0.34 |
| Stress | Regression | Use Time | 1 | 29 | -0.16 | -0.14 |
| Neglect* | Regression | Mood | 1 | 29 | -0.17 | -0.14 |
| Neglect* | Regression | Stress | 1 | 25 | 0.13 | 0.13 |
| Mood | Regression | Neglect | 1 | 22 | -0.10 | -0.12 |
| Use Time* | Regression | Stress | 1 | 22 | -0.11 | -0.12 |
| Temptation | Regression | Neglect | 2 | 20 | -1.99 | -1.68 |
| Neglect* | Regression | Stress | 2 | 16 | 0.28 | 0.28 |
| Temptation | Covariance | Neglect | 2 | 16 | -3.00 | -1.36 |
| Relief* | Regression | Stress | 1 | 15 | 0.09 | 0.09 |
| Mood | Regression | Relief | 1 | 13 | 0.08 | 0.09 |
| Mood | Regression | Use Time | 1 | 11 | -0.09 | -0.09 |
| Pleasure* | Regression | Stress | 2 | 11 | -0.20 | -0.20 |
| Pathological use group | | | | | | |
| Outcome | Relation | Predictor | Level | MI | EPC | SEPC |
| Temptation | Covariance | Pleasure | 1 | 67 | 2.41 | 0.75 |
| Temptation | Regression | Pleasure | 1 | 63 | 0.92 | 0.84 |
| Pleasure* | Regression | Mood | 1 | 63 | 0.17 | 0.17 |
| Mood | Regression | Pleasure | 1 | 41 | 0.15 | 0.15 |
| Temptation | Covariance | Neglect | 1 | 33 | -2.09 | -0.58 |
| Neglect* | Regression | Stress | 1 | 27 | 0.12 | 0.11 |
| Temptation | Regression | Neglect | 1 | 27 | -0.53 | -0.58 |
| Temptation | Regression | Neglect | 2 | 26 | -1.59 | -1.51 |
| Pleasure* | Regression | Stress | 1 | 24 | -0.09 | -0.10 |
| Neglect* | Regression | Stress | 2 | 24 | 0.33 | 0.31 |
| Temptation | Covariance | Neglect | 2 | 24 | -3.21 | -1.22 |
| Stress | Regression | Use Time | 1 | 22 | -0.15 | -0.12 |
| Neglect* | Regression | Mood | 1 | 20 | -0.12 | -0.10 |
| Use Time* | Regression | Stress | 1 | 19 | -0.07 | -0.09 |
| Mood | Regression | Use Time | 1 | 17 | -0.12 | -0.11 |
| Pleasure* | Regression | Mood | 2 | 16 | 0.23 | 0.19 |
| Mood | Regression | Neglect | 1 | 12 | -0.07 | -0.09 |
| *Relevant to the decision regarding model adjustments | | | | | | |
