# Supplementary material for: Temptation as a key driver between affective states and usage outcomes of problematic usage of the Internet: A 14-day ambulatory assessment study
Source: PLoS One. 2026 Jul 29;21(7):e0352776. doi: 10.1371/journal.pone.0352776 (PMC13419235; doi:10.1371/journal.pone.0352776)
Supplement: S9 Table — (DOCX) [file pone.0352776.s009.docx]

| **Table S9. Multigroup multilevel structure equation model: regression effects of the initial model.** | | | | | | | | | | | | | | | | |
| --- | --- | --- | --- | --- | --- | --- | --- | --- | --- | --- | --- | --- | --- | --- | --- | --- |
| Outcome | Predictor | Non-problematic use group | | | | | Risky use group | | | | | Pathological use group | | | | |
| Level 1 (within-person) | | *b* | *SE* | *z* | *p* | *β* | *b* | *SE* | *z* | *p* | *β* | *b* | *SE* | *z* | *p* | *β* |
| Temptation | Stress | 0.04 | 0.02 | 1.81 | .070 | 0.04 | 0.02 | 0.03 | 0.57 | .569 | 0.02 | 0.11 | 0.02 | 4.50 | <.001 | 0.11 |
|  | Mood | -0.11 | 0.03 | -3.97 | <.001 | -0.09 | -0.07 | 0.03 | -2.22 | .027 | -0.06 | -0.19 | 0.03 | -7.03 | <.001 | -0.17 |
| Use Time | Temptation | 0.28 | 0.01 | 21.42 | <.001 | 0.43 | 0.35 | 0.02 | 17.24 | <.001 | 0.41 | 0.35 | 0.02 | 22.62 | <.001 | 0.47 |
| Neglect | Use Time | 0.40 | 0.03 | 13.10 | <.001 | 0.28 | 0.36 | 0.03 | 11.62 | <.001 | 0.29 | 0.60 | 0.03 | 19.59 | <.001 | 0.41 |
| Pleasure | Use Time | 0.36 | 0.03 | 11.83 | <.001 | 0.25 | 0.21 | 0.03 | 7.37 | <.001 | 0.19 | 0.28 | 0.03 | 10.22 | <.001 | 0.23 |
| Relief | Use Time | 0.32 | 0.03 | 10.55 | <.001 | 0.23 | 0.14 | 0.03 | 4.64 | <.001 | 0.12 | 0.30 | 0.03 | 10.64 | <.001 | 0.24 |
| Level 2 (between-person) | | *b* | *SE* | *z* | *p* | *β* | *b* | *SE* | *z* | *p* | *β* | *b* | *SE* | *z* | *p* | *β* |
| Temptation | Stress | 0.30 | 0.07 | 4.27 | <.001 | 0.24 | 0.28 | 0.08 | 3.36 | .001 | 0.23 | 0.30 | 0.08 | 3.95 | <.001 | 0.27 |
|  | Mood | 0.09 | 0.08 | 1.13 | .258 | 0.06 | 0.04 | 0.10 | 0.36 | .717 | 0.03 | -0.10 | 0.09 | -1.11 | .268 | -0.08 |
| Use Time | Temptation | 0.08 | 0.03 | 2.42 | .015 | 0.13 | 0.07 | 0.06 | 1.09 | .276 | 0.07 | -0.04 | 0.06 | -0.68 | .494 | -0.05 |
| Neglect | Use Time | 0.26 | 0.06 | 4.44 | <.001 | 0.28 | 0.07 | 0.07 | 1.03 | .305 | 0.08 | 0.13 | 0.08 | 1.72 | .085 | 0.13 |
| Pleasure | Use Time | 0.38 | 0.08 | 5.09 | <.001 | 0.30 | 0.21 | 0.06 | 3.39 | .001 | 0.25 | -0.02 | 0.07 | -0.23 | .820 | -0.02 |
| Relief | Use Time | 0.23 | 0.09 | 2.68 | .007 | 0.16 | 0.17 | 0.07 | 2.26 | .024 | 0.16 | 0.03 | 0.07 | 0.35 | .726 | 0.03 |
| Note. The within-person predictor variables are centered within cluster. The between-person predictor variables are grand mean centered. | | | | | | | | | | | | | | | | |
