# Supplementary material for: Temptation as a key driver between affective states and usage outcomes of problematic usage of the Internet: A 14-day ambulatory assessment study
Source: PLoS One. 2026 Jul 29;21(7):e0352776. doi: 10.1371/journal.pone.0352776 (PMC13419235; doi:10.1371/journal.pone.0352776)
Supplement: S10 Table — (DOCX) [file pone.0352776.s010.docx]

| **Table S10. Multigroup multilevel structure equation model: regression effects of the adjusted model.** | | | | | | | | | | | | | | | | |
| --- | --- | --- | --- | --- | --- | --- | --- | --- | --- | --- | --- | --- | --- | --- | --- | --- |
| Outcome | Predictor | Non-problematic use group | | | | | Risky use group | | | | | Pathological use group | | | | |
| Level 1 (within-person) | | *b* | *SE* | *z* | *p* | *β* | *b* | *SE* | *z* | *p* | *β* | *b* | *SE* | *z* | *p* | *β* |
| Temptation | Stress | 0.04 | 0.02 | 1.81 | .070 | 0.04 | 0.02 | 0.03 | 0.57 | .569 | 0.02 | 0.11 | 0.02 | 4.50 | <.001 | 0.11 |
|  | Mood | -0.11 | 0.03 | -3.97 | <.001 | -0.09 | -0.07 | 0.03 | -2.22 | .027 | -0.06 | -0.19 | 0.03 | -7.03 | <.001 | -0.17 |
| Use Time | Temptation | 0.28 | 0.01 | 19.71 | <.001 | 0.40 | 0.35 | 0.02 | 15.91 | <.001 | 0.38 | 0.35 | 0.02 | 21.21 | <.001 | 0.44 |
| Neglect | Temptation | 0.30 | 0.02 | 15.47 | <.001 | 0.32 | 0.29 | 0.03 | 11.06 | <.001 | 0.28 | 0.43 | 0.02 | 18.31 | <.001 | 0.39 |
| Pleasure | Temptation | 0.26 | 0.02 | 13.71 | <.001 | 0.29 | 0.27 | 0.02 | 11.36 | <.001 | 0.28 | 0.22 | 0.02 | 10.65 | <.001 | 0.24 |
| Relief | Temptation | 0.25 | 0.02 | 13.00 | <.001 | 0.28 | 0.29 | 0.03 | 11.35 | <.001 | 0.28 | 0.30 | 0.02 | 14.63 | <.001 | 0.32 |
| Level 2 (between-person) | | *b* | *SE* | *z* | *p* | *β* | *b* | *SE* | *z* | *p* | *β* | *b* | *SE* | *z* | *p* | *β* |
| Temptation | Stress | 0.30 | 0.07 | 4.27 | <.001 | 0.24 | 0.28 | 0.08 | 3.35 | .001 | 0.23 | 0.30 | 0.08 | 3.95 | <.001 | 0.27 |
|  | Mood | 0.09 | 0.08 | 1.13 | .259 | 0.06 | 0.04 | 0.10 | 0.36 | .717 | 0.03 | -0.10 | 0.09 | -1.11 | .267 | -0.08 |
| Use Time | Temptation | 0.08 | 0.04 | 2.07 | .038 | 0.13 | 0.07 | 0.07 | 1.01 | .312 | 0.07 | -0.02 | 0.06 | -0.38 | .702 | -0.03 |
| Neglect | Temptation | 0.27 | 0.04 | 7.27 | <.001 | 0.45 | 0.38 | 0.06 | 6.32 | <.001 | 0.45 | 0.56 | 0.06 | 9.20 | <.001 | 0.58 |
| Pleasure | Temptation | 0.50 | 0.04 | 11.74 | <.001 | 0.61 | 0.31 | 0.06 | 5.18 | <.001 | 0.37 | 0.50 | 0.06 | 8.90 | <.001 | 0.57 |
| Relief | Temptation | 0.56 | 0.05 | 11.82 | <.001 | 0.60 | 0.47 | 0.07 | 7.07 | <.001 | 0.48 | 0.60 | 0.05 | 11.04 | <.001 | 0.65 |
| Note. The within-person predictor variables are centered within cluster. The between-person predictor variables are grand mean centered. | | | | | | | | | | | | | | | | |
